# Zero and Ultra-Short Echo Time Sequences at 3-Tesla Can Accurately Depicts the Normal Anatomy of the Human Achilles Tendon Enthesis Organ In Vivo

**DOI:** 10.3390/jcm14155251

**Published:** 2025-07-24

**Authors:** Amandine Crombé, Benjamin Dallaudière, Marie-Camille Bohand, Claire Fournier, Paolo Spinnato, Nicolas Poursac, Michael Carl, Julie Poujol, Olivier Hauger

**Affiliations:** 1 Department of Musculoskeletal Imaging, Pellegrin University Hospital CHU Bordeaux, 33076 Bordeaux, France; 2 SARCOTARGET Team, Inserm, UMR1312, BRIC, Bordeaux Institute of Oncology, University of Bordeaux, 33076 Bordeaux, France; 3 Centre d’Imagerie Ostéo-articulaire, Clinique du Sport de Bordeaux-Mérignac, 2 rue Négrevergne, 33700 Mérignac, France; 4 Centre de Résonance Magnétique des Systèmes Biologiques (CRMSB), UMR 5536, CNRS, Université de Bordeaux, 146 rue Léo Saignat, 33076 Bordeaux, France; 5 Diagnostic and Interventional Radiology, IRCCS, Istituto Ortopedico Rizzoli, 40136 Bologna, Italy; 6 Department of Rheumatology, Pellegrin University Hospital CHU Bordeaux, 33000 Bordeaux, France; 7 GE HealthCare, Chicago, IL 60661, USA; 8 GE HealthCare, 78530 Buc, France

**Keywords:** Achilles tendon, magnetic resonance imaging, enthesis, ultra-short echo time, zero echo time

## Abstract

**Background/Objectives**: Accurate visualization of the Achilles tendon enthesis is critical for distinguishing mechanical, degenerative, and inflammatory pathologies. Although ultrasonography is the first-line modality for suspected enthesis disease, recent technical advances may expand the role of magnetic resonance imaging (MRI). This study evaluated the utility of ultra-short echo time (UTE) and zero echo time (ZTE) sequences versus proton density-weighted imaging (PD-WI) for depicting the enthesis organ in healthy volunteers. **Methods:** In this institutional review board (IRB)-approved prospective single-center study, 50 asymptomatic adult volunteers underwent 3-Tesla hindfoot MRI with fat-suppressed PD-WI, UTE, and ZTE between 2018 and 2023. Four radiologists assessed image quality, signal-to-noise ratio, visibility, and abnormal high signal intensities (SIs) of the periost, sesamoid, and enthesis fibrocartilages (PCa, SCa, and ECa, respectively). Statistical tests included Chi-square, McNemar, paired Wilcoxon, and Benjamini–Hochberg adjustments for multiple comparisons. **Results**: The median age was 36 years (range: 20–51); 58% women were included. PD-WI and ZTE sequences were always available while UTE was unavailable in 24% of patients. PD-WI consistently failed to concomitantly visualize all fibrocartilages. ZTE and UTE visualized all fibrocartilages in 72% and 92.1% of volunteers, respectively, with significant differences favoring ZTE and UTE over PD-WI (*p* < 0.0001) and UTE over ZTE (*p* = 0.027). Inter-rater agreement exceeded 80% except for SCa on ZTE (68%, 95%CI: 53.2–80.1). Abnormal SCa findings in asymptomatic patients were more frequent with UTE (23.7%) and ZTE (34%) than with PD-WI (2%) (*p* = 0.0045). **Conclusions**: At 3-Tesla, UTE and ZTE sequences reliably depict the enthesis organ of the Achilles tendon, outperforming PD-WI. However, the high sensitivity of these sequences also presents challenges in interpretation.

## 1. Introduction

The Achilles tendon is a biomechanically complex structure composed of a posterior collagenous component and an anterior fibrocartilaginous structure known as the ‘enthesis organ’ [1], which plays a critical role in dissipating stress at the tendon–bone interface [2,3]. This structure comprises the periosteal cartilage (PCa), the sesamoid cartilage (SCa), the enthesis cartilage (ECa), the retrocalcaneal bursa, and a synovial-lined fat pad [4]. Specifically, the ECa is the most distal fibrocartilage located at the tendon–bone interface, while the SCa and the PCa extend the synovial lining of the bursa, protecting the tendon during dorsiflexion [3,5].

The Achilles tendon is one of the most frequently injured tendons in the human body [6]. Remarkably, it has been termed the ‘premier organ’ by rheumatologists due to its early involvement in seronegative spondyloarthropathies, even in asymptomatic patients [3,4]. Understanding the enthesis organ would be pivotal for the diagnostic and therapeutic management of spondyloarthropathy patients [7] and to differentiate mechanical, inflammatory, and non-pathological findings.

The non-invasive assessment of Achilles tendon and enthesis alterations in clinical practice relies on color Doppler ultrasonography (CDUS) and magnetic resonance imaging (MRI) [8]. CDUS is the first-line imaging tool due to its ability to detect vascular signals linked to active inflammation, but its performance is highly operator-dependent and can be limited in obese or anechoic patients. MRI offers a standardized protocol and allows a comprehensive evaluation of the hindfoot, including tendons, joints, and bone marrow. However, the evaluation of structures with very short transverse relaxation times (T2), especially tendons, ligaments, entheses, and cartilages, poses challenges for conventional MRI like fat-suppressed proton density-weighted imaging (PD-WI) and T2-weighted imaging (T2-WI). Ultra-short echo time (UTE) and zero echo time (ZTE) sequences have been developed to address these limitations [9,10]. These techniques utilize specialized acquisition and reconstruction methods to achieve echo times (TEs) approaching or equal to 0 ms, enabling the visualization of structures with very short T2. The most current implementation of ZTE into commercial scanners is now based on a rotating ultra-fast imaging sequence (RUFIS) strategy [11], which employs 3D radial center-out k-space encoding [12,13]. Furthermore, it employs non-selective volume excitation with hard radiofrequency pulse to achieve consistent excitation independent of the gradient readout changing over time and to reduce the dead-time gap to sample center data [14].

Previous studies investigating UTE sequences have been limited to small cohorts of cadavers, volunteers, or symptomatic patients, often fewer than 10 subjects [15,16,17,18]. For instance, Robson et al. demonstrated strong correspondence between UTE images and the histological structure of the Achilles tendon enthesis organ in a cohort of four patients and four volunteers [16]. The findings were later corroborated on a 7-Tesla MRI scanner in five volunteers and one patient with additional fat suppression [17]. Both studies demonstrate the need for fat-suppressed UTE to better characterize the enthesis. However, these studies often employed lengthy acquisition times, either with multi-echo UTE sequence needing specific post-processing or a highly resolved UTE sequence taking up to 18 min, limiting its practicality in clinical routine.

Despite this progress, there is a lack of comprehensive studies depicting the Achilles tendon enthesis organ in vivo in larger, more representative cohorts of healthy adult volunteers using both UTE and ZTE. Additionally, the normal anatomical variations of the enthesis organ on UTE and ZTE in asymptomatic individuals remain unexplored. Addressing these gaps would not only refine diagnostic accuracy but also help mitigate false positives in future clinical research involving patients.

Thus, our main hypothesis was that UTE and ZTE would improve the entire visibility of the Achilles enthesis organ compared to conventional proton-density weighted imaging. Therefore, the primary aim of this study was to evaluate in a clinical setting the utility of innovative UTE and ZTE sequences developed by General Electrics (GE) Healthcare compared to PD-WI in visualizing the enthesis organ of the Achilles tendon in a prospective single-center cohort of 50 healthy adult volunteers at 3-Tesla. Secondary objectives included assessing inter- and intra-observer agreements, comparing image quality, and identifying abnormal findings in the PCa, SCa, and ECa in asymptomatic volunteers.

## 2. Materials and Methods

### 2.1. Study Design

This prospective, observational, single-center study was approved by the ethical committee from Bordeaux University Hospital (approval number: ID-RCB-2017-A01543–50; clinical trial number: NCT03287596, initially submitted in September 2017 on the following registry: https://clinicaltrials.gov/study/NCT03287596?tab=history) (accessed on 20 May 2025).

All patients gave their written informed consent to participate in the study. The study was conducted in accordance with Good Clinical Practice guidelines, applicable regulations, and the principles of the Declaration of Helsinki. No funding was needed for this study.

The study was designed to include 50 consecutive healthy adults from our institution between April 2018 and January 2023. Healthy controls were recruited through advertisements placed on hospital and university bulletin boards and collaboration with local health organizations. The screening process included medical history reviews and clinical examination to ensure they met the study’s definition of ‘healthy’ and had no MRI contraindications. None of the patients had a history of Achilles tendon or enthesis pathology, and none had ever received medication administered near the Achilles tendon enthesis.

Inclusion criteria were age ≥ 18 years and no medical history of calcaneal tendinopathy, plantar support and pain, calcaneal surgery, and axial or peripheral rheumatism.

Exclusion criteria were pregnant or lactating volunteers, known active coronaropathy disease, septic arthritis, contraindications to the realization of an MRI, and patients under justice protection.

The main study endpoint was the visibility of the normal anatomical areas of the Achilles tendon enthesis assessed on all sequences.

### 2.2. Clinical Data Collection

Initial clinical data collection comprised patient age, sex, height, weight, body mass index (BMI, calculated as weight in kilograms divided by height in meters squared), and medical history.

In October 2024 (i.e., 22 months after last patient inclusion), all patients were contacted by phone by a resident in radiology (M.C.B, with one year of experience in musculoskeletal imaging) with a standardized questionnaire including (i) whether plantar symptoms had occurred after the MRI, and in cases of positive answers, (ii) how many time after, (iii) whether the heel pain followed an inflammatory or mechanical pattern, (iv) which diagnosis was confirmed and by whom, and (v) which therapeutic management was proposed. At least three phone calls were attempted on three different days to volunteers who did not answer.

### 2.3. MRI Acquisition

MR examinations were all performed on the same 3-Tesla magnet (Discovery MR750 W, GE HealthCare, Madison, WI, USA). The left or right side of the hindfoot was randomly chosen.

The protocol was standardized, centered on the distal enthesis of the Achilles tendon and the posterior process of the calcaneus, and comprised one sagittal T1-WI, one sagittal and one axial fat-suppressed PD-WI, one sagittal and one axial fat-suppressed UTE sequence, and one sagittal and one axial fat-suppressed ZTE sequence. Both UTE and ZTE sequences were shared through a research partnership with GE HealthCare and optimized with high in-plane spatial resolution (0.30 × 0.30 mm^2^) for better visualization of enthesis components. Slice thickness was 1.3 mm for both sequences to increase the signal-to-noise ratio (SNR) and keep a reasonable acquisition time (Table 1).

Moreover, UTE and ZTE work-in-progress sequences were characterized by several optimizations summarized in Appendix A.

Examinations were acquired with the flexible 16-channel coil adapted to ankle imaging. The total duration of the examination was 40 min.

### 2.4. MRI Analysis

First, MR examinations were reviewed individually by two seniors in musculoskeletal imaging (A.C. and O.H., with 25 and 5 years of experience, respectively), blinded to any patient and study information and to the other radiologist’s reading.

**Figure 1:**
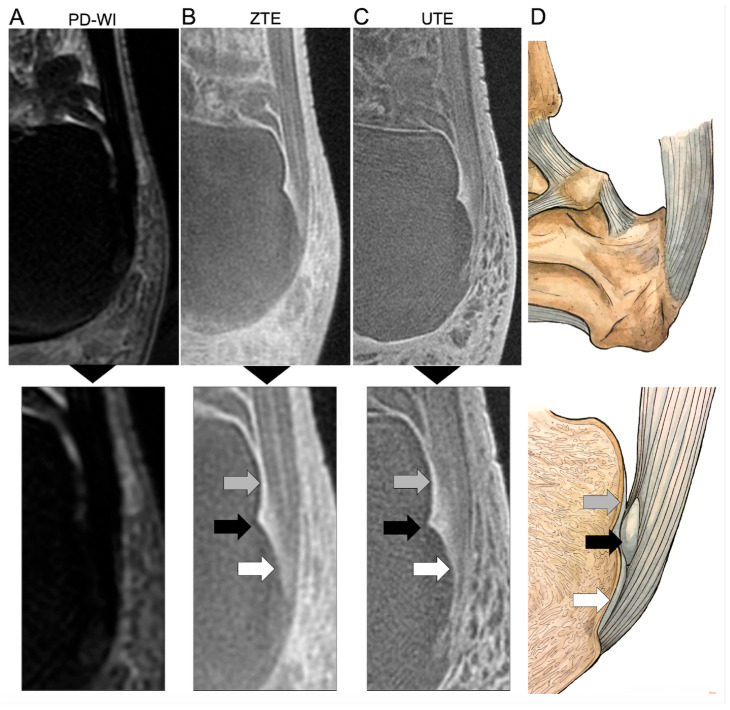
Assessment of the normal hindfoot and Achilles tendon ‘enthesis organ’ on proton density weighted imaging (PD-WI) ((**A**), upper panel) with magnification (lower panel), zero echo time imaging (ZTE) (**B**), and ultrashort echo time imaging (UTE) (**C**) MRI sequences and a corresponding representation of the real anatomy (**D**). The enthesis organ is made of three anterior fibrocartilages, all imperceptible with PD-WI, but demonstrating high signal intensity on UTE and ZTE sequences. The gray arrow shows the periost fibrocartilage (PCa) as a thin line at the posterior and superior surface of the superior tuberosity of the calcaneum. The black arrow shows the sesamoid fibrocartilage (SCa) as a ground-glass area within a notch at the middle of the posterior surface of the calcaneus. Lastly, the white arrow shows the enthesis fibrocartilage (ECa) at the interface of the tendon itself and the bone. The stripes on the Achilles tendon correspond to the endotenon (with high signal intensity on UTE and ZTE) and the tendon fascicle (with lower signal).

For each type of MR sequence (PD-WI, UTE, and ZTE), they assessed (i) whether each of the three components of the Achilles enthesis was clearly individualized or not, namely the ECa, SCa, and PCa. They concluded whether (ii) the entire anatomy was clearly distinguishable. Figure 1 represents this normal anatomy and its correspondence on UTE and ZTE. Next, for each of these fibrocartilages (ECa, SCa, and PCa) and for each sequence type, they indicated (iii) whether they considered the aspect normal, pathological, or doubtful.

Finally, they concluded whether (iv) they considered the Achilles enthesis as normal, pathological, or doubtful.

Two months after her first reading, one of the radiologists (A.C.) reviewed the full MRI dataset using the same analysis grid to estimate the intra-observer reproducibility of these radiological variables.

Finally, a consensus reading for all of these variables was performed 3 months later by M.C.B., O.H., A.C., and B.D. (another senior in musculoskeletal imaging with 15 years of experience). Additionally, this final consensus reading also assessed (v) the quality of the PD-WI, UTE, and ZTE sequences according to a 3-point ordinal scale (i.e., categorized as: poor [i.e., non-diagnostic], intermediate [i.e., imperfect but can be analyzed] and good quality); (vi) the mean and standard deviation (SD) of the SI (either on sagittal UTE or sagittal ZTE) measured in circular 2D regions-of-interest (ROIs) of at least 1 cm^2^ placed on the Achilles tendon, the medulla of the calcaneus, and the surrounding noise in order to estimate the SNR according to the following formula: SNR_tendon_ = Mean[SI_tendon_]/SD[SI_noise_] and SNR_calcaneus_ = Mean[SI_calcaneus_]/SD[SI_noise_] (i.e., mean signal intensity value in the ROI of the structure of interest, divided by the SD of the noise ROI); and (vii) the presence of enthesophytes and calcifications in the Achilles enthesis.

A.C. (as well as the consensus reading) started by reading PD-WI, UTE, and ZTE for the first 25 patients, then PD-WI, ZTE, and UTE for the last 25 patients. O.H. started by reading PD-WI, ZTE, and UTE for the first 25 patients, then PD-WI, UTE, and ZTE for the last 25 patients.

**Figure 2:**
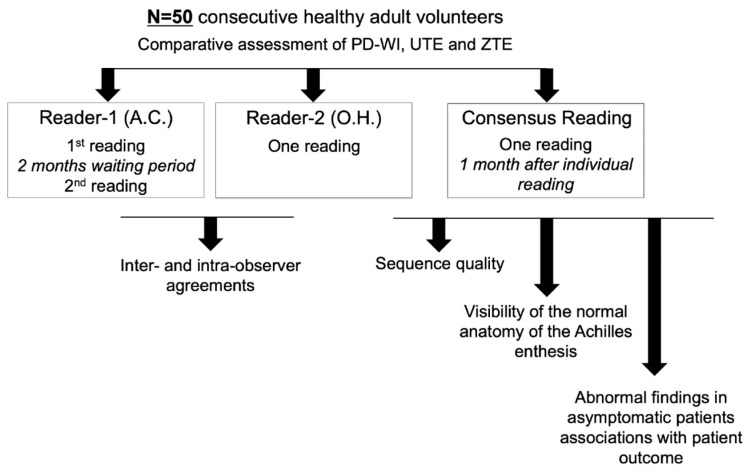
Statistical workflow. Abbreviations: PD–WI: proton density weighted imaging; UTE: ultrashort echo time imaging; ZTE: zero echo time imaging (ZTE).

Figure 2 represents the study workflow illustrating the different readings performed on the dataset and the subsequent analysis performed on these readings (i.e., the assessment of intra- and inter-observer agreements, sequent quality, visibility of the normal anatomy of the Achilles enthesis organ, and observations of abnormal findings in asymptomatic patients).

### 2.5. Statistical Analysis

Statistical analyses were performed with R (v4.1.1, Vienna, Austria). All tests were two-tailed. A *p*-value < 0.05 was considered significant. Adjustments for multiple comparisons were performed using the Benjamini–Hochberg method.

*Quality assessment*. Associations between quality score and sequence type were investigated with the Chi-square test. Associations between SNRs and sequence type (UTE and ZTE) were investigated with paired Wilcoxon tests after verifying the non-normality of the SNR.

*Visibility of the normal anatomy of the Achilles enthesis*. The rates of volunteers with visible ECa, SCa, PCa, and whole anatomy were compared between the PD-WI, UTE, and ZTE sequences using Chi-square and McNemar tests.

*Inter- and intra-observer agreements*. The percentages of absolute agreement with 95% confidence intervals (95%CI) between readings were estimated using the binom.test R function.

Raw data and R scripts from the study are available from the corresponding author upon reasonable request.

## 3. Results

### 3.1. Characteristics of the Study Population

The median age was 36 years (IQR: 26–38, range: 20–50), and 29/50 (58%) women were included. No patient declared the use of any medical treatment, including any treatment at risk of Achilles tendon lesion (i.e., statins, anti-aromatase, steroids, or quinolone). The median BMI was 21.8 kg m^−2^ (IQR: 20.8–24.1, range: 16.3–33.9) with 8/50 (16%) overweight patients and 2/50 (4%) obese patients. No anatomical variant was observed on the ankle and posterior foot.

### 3.2. Quality Assessment

DP-WI and ZTE sequences were available for all patients while UTE sequences were unavailable for 12/50 (24%) patients due to fat-suppression failure (n = 2/12, 16.7%), reconstruction error, and transfer issues to the PACS (n = 10/12, 83.3%).

Table A1 shows the comparative quality assessment of the UTE and ZTE sequences in the 38 patients with both UTE and ZTE sequences. The SNR was significantly higher using the ZTE sequence compared to the UTE sequence for both the Achilles tendon and the calcaneus (*p* < 0.0001 and *p* = 0.0001, respectively). However, the quality scores were significantly better for the UTE sequence, with 92.1% (35/38) acquisitions rated as high quality with UTE versus 42.1% (16/38) with ZTE (*p* < 0.0001).

In the consensual reading, the preferred sequences in terms of image quality were, in descending order: UTE (26/38, 68.4%), ZTE (5/38, 13.2%), and both equally (1/38, 2.6%). The UTE and ZTE sequences were systematically preferred to the PD-WI sequences.

### 3.3. Visibility of the Normal Anatomy

Figure 1 shows an example of the entire anatomy of the Achilles tendon correctly identified on UTE and ZTE sequences but not on PD-WI. Table 2 summarizes the assessment of the Achilles enthesis anatomy with UTE, ZTE, and PD-WI.

**Figure 3:**
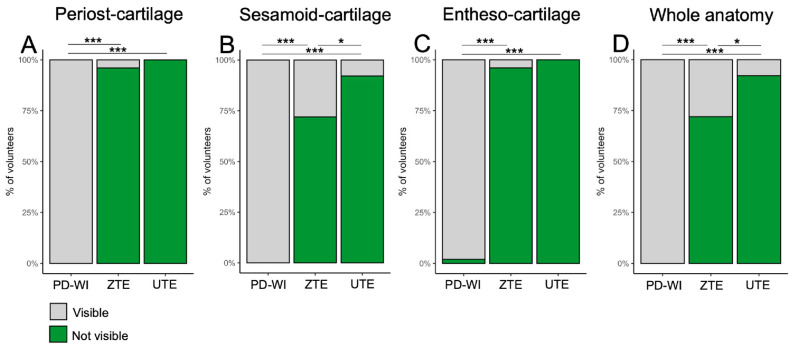
Visibility of each fibrocartilage of the Achilles tendon enthesis organ, i.e., the periost fibrocartilage (**A**), the sesamoid fibrocartilage (**B**), the enthesis fibrocartilage (**C**), and subsequent whole anatomy (**D**) depending on the MR-sequence, according to the consensus reading. Abbreviation: PD-WI: proton density weighted imaging. * *p* < 0.05, *** *p* < 0.001.

The PCa was correctly seen on 0/50 (0%) patients using PD-WI, 48/50 (96%) patients using ZTE, and 38/38 (100%) using UTE (Figure 3A). This proportion was significantly higher with UTE versus PD-WI (*p* < 0.0001) and ZTE versus PD-WI (*p* < 0.0001) but not between UTE and ZTE (*p* = 1).

The SCa was correctly seen on 0/50 (0%) patients using PD-WI, 36/50 (72%) patients using ZTE, and 35/38 (92.1%) using UTE (Figure 3B). This proportion was significantly higher with UTE versus PD-WI (*p* < 0.0001), ZTE versus PD-WI (*p* < 0.0001), and UTE versus ZTE (*p* = 0.0269).

The ECa was correctly seen on 1/50 (2%) patients using PD-WI, 48/50 (96%) patients using ZTE, and 38/38 (100%) using UTE (Figure 3C). This proportion was significantly higher with UTE versus PD-WI (*p* < 0.0001) and ZTE versus PD-WI (*p* < 0.0001) but not between UTE and ZTE (*p* = 1)

Finally, the entire anatomy of the enthesis was correctly identified on 0/50 (0%) patients using PD-WI, 36/50 (72%) patients using ZTE, and 35/38 (92.1%) using UTE (Figure 3D). This proportion was significantly higher with UTE versus PD-WI (*p* < 0.0001), ZTE versus PD-WI (*p* < 0.0001), and UTE versus ZTE (*p* = 0.0269).

### 3.4. Inter- and Intra-Observer Agreements

Inter- and intra-observer agreements are summarized in Table 3. Regarding the PD-WI sequence, the visibility of the three segments of the enthesis was weak across all readings with strong inter- and intra-rater agreements (>80%) for the SCa, ECa, and the whole anatomy, except for the PCa (inter-observer agreement: 56% [95%CI: 41.3–69.7]; intra-observer agreement: 70% [95%CI: 55.2–81.7]).

Regarding the UTE and ZTE sequences, they enabled the visibility of the enthesis anatomy in most patients with strong inter- and intra-rater agreements except for the SCa using ZTE (inter-rater agreement: 68% [95%CI: 53.2–80.1]) and subsequently the entire anatomy (inter-rater agreement: 66% [95%CI: 51.1–78.4]).

### 3.5. Abnormal Findings in Healthy Volunteers and Correlation with Patient Outcome

Calcifications and ossifications in the Achilles enthesis were seen in the same eight patients, regardless of MR sequence.

Table 4 shows the rates of doubtful and pathological findings in healthy volunteers for the three sequences and three locations.

Regarding the PCa, there were no abnormal findings using PD-WI; however, the radiologists described 3/38 (7.9%) as doubtful of pathological findings with the UTE sequence and 5/50 (10%) with ZTE. The rate of false positive findings was not significantly different across the three sequences (*p* = 0.2818).

Regarding the SCa, there was one abnormal finding using PD-WI; however, the radiologists described 5/38 (13.2%) as doubtful of pathological findings with the UTE sequence and 9/50 (18%) with ZTE. The rate of false positive findings was significantly different across the three sequences (*p* = 0.0045). In paired post-hoc adjusted analyses, there was no significant difference in the rate of false positive findings with UTE compared to PD-WI, ZTE compared to PD-WI (*p* = 0.1944 and *p* = 0.1944, respectively), and between UTE and ZTE (*p* = 0.9279).

Regarding the ECa, there was 1/50 (2%) abnormal finding using PD-WI; however, the radiologists described 8/38 (23.7%) as doubtful of pathological findings with the UTE sequence and 17/50 (34%) with ZTE. The rate of false positive findings was not significantly different across the three sequences (*p* = 0.0746).

Regarding follow-up questionnaires, 43/50 (86%) answered them. One volunteer declared the appearance of hindfoot pain about one year after his MRI, which revealed, at that time, abnormal findings on UTE and ZTE sequences (but not on PD-WI) at PCa and SCa locations.

**Figure 4:**
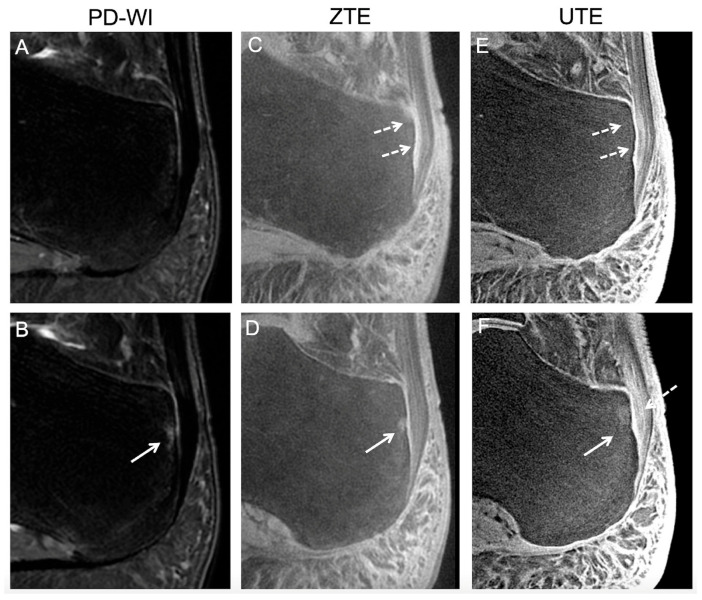
Abnormal findings in an asymptomatic volunteer involving the periost and the sesamoid fibrocartilages reported during the consensus reading. The healthy volunteer was a 29-year-old male. Two consecutive slices are shown for each sequence, i.e., proton density weighted imaging (PD-WI) (**A**,**B**), ZTE (**C**,**D**), and UTE (**E**,**F**). Abnormal findings involving the periost cartilage on UTE and ZTE sequences (but not on PD-WI) corresponded to an ill-defined thick area with high signal intensity (white dashed arrows). Abnormal findings involving the sesamoid cartilage, seen on all sequences, corresponded to a focal bone edema (white continuous arrows). However, the patient did not declare hindfoot pain during follow-up.

**Figure 5:**
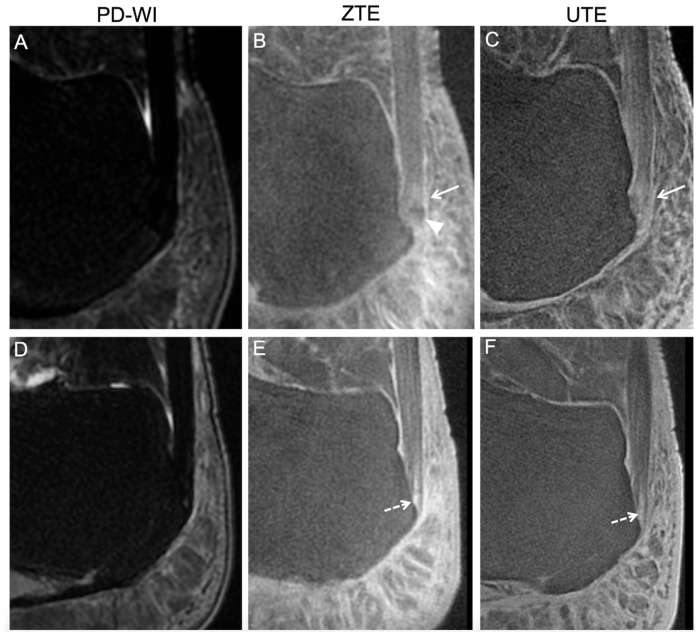
Abnormal findings in asymptomatic volunteers involving the enthesis fibrocartilage reported during the consensus reading. The healthy volunteers were a 42-year-old overweight woman (top row) and a 51-year-old man (bottom row). On proton density weighted imaging (PD-WI), no abnormal findings were described for either patient (**A**,**D**). However, the first patient demonstrated a focal ossification (white arrow head) and high signal intensities on ZTE and UTE (white arrows) (**B**,**C**) at the location of the enthesis fibrocartilage. The second patient displayed a linear fissure at the enthesis fibrocartilage on ZTE (**E**) and UTE (**F**). Later, during the follow-up, he declared hindfoot pain.

Figure 4 and Figure 5 illustrate the MRI findings described as pathological. Regarding the five patients with abnormal findings on the ECa with both UTE and ZTE, the retrospective reading confirmed the presence of high SIs of the ECA and Achilles tendinous fibers surrounding enthesophytes (Figure 4). Regarding the two volunteers with abnormal findings on the SCa and PCa on both UTE and ZTE sequences (including the volunteer who developed hindfoot pain one year later), the retrospective reading confirmed the presence of focal erosion at the SCa and high SIs of the PCa and its surrounding fibers (Figure 5).

## 4. Discussion

Accurate MRI identification of the Achilles tendon enthesis organ is essential for distinguishing disease-specific pathological patterns. This study evaluated prototype UTE and ZTE sequences against PD-WI in 50 healthy adult volunteers. UTE and ZTE significantly improved visualization of the PCa, SCa, and ECa compared to PD-WI. Moreover, while ZTE demonstrated a higher SNR compared to UTE, UTE provided a more accurate and reproducible assessment of the normal anatomy of the Achilles tendon enthesis, especially regarding SCa.

Prior studies have already emphasized the interest of UTE sequencing to explore the Achilles tendon, but in smaller cohorts, without including ZTE for comparisons and requiring scanning times incompatible with clinical practice. In agreement with prior studies [15,16,17], our results demonstrated the strong improvement of both UTE and ZTE for the in vivo visualization of the three fibrocartilages constituting the Achilles enthesis. The PCa, SCa, and ECa were concomitantly observed in 92.1% of volunteers using UTE and 72% volunteers using ZTE against 0% of volunteers using PD-WI. Furthermore, UTE demonstrated better image quality, with 92.1% of acquisitions rated as high quality compared to 42.1% for ZTE. Additionally, UTE sequences provided superior visualization of the SCa (92.1% versus 72% for ZTE) and were preferred by readers in 68.4% of cases compared to 13.2% for ZTE. It must be emphasized that the accurate depiction of normal and pathological SCa would be crucial in studies involving patients with seronegative spondyloarthropathies, as this area is often affected in early disease stages. Li et al. observed abnormal vascularization in the peri-sesamoidal area of spondyloarthropathy patients using CDUS [19]. In a murine model of spondyloarthropathy, Dallaudière et al. demonstrated that SCa was affected by fibrillar disorganization and macrophagic infiltration in the early phase, which later spread to the ECa and ultimately contributed to bone and spur production [20]. These findings suggest that identifying subtle pathological features in the SCa using UTE or ZTE sequences could potentially indicate patients at higher risk of spondyloarthropathy or disease relapse, possibly before symptom onset. This underscores the potential of these advanced sequences in the early detection and monitoring of spondyloarthropathies.

However, ZTE sequences exhibited a higher SNR for both the Achilles tendon and calcaneus. These differences may be attributed to the specific technical characteristics of each sequence, with ZTE offering reduced sensitivity to gradient imperfections.

The high inter- and intra-observer agreements (>80% for most structures) observed for both UTE and ZTE underscore their reliability in evaluating the Achilles enthesis. This contrasts with the poor visibility and higher variability seen with PD-WI. Moreover, we observed more variable rates of visibility (and lower agreements) for the PCa on PD-WI, which could be attributed to the confusion between very thin retrocalcaneal bursa adjacent to the PCa and the PCa itself. Similarly, the visualization of the SCa seemed more challenging with ZTE compared to UTE, due to its smaller size, intermediate ground-glass signal, and anatomical variations.

An intriguing finding was the detection of abnormalities in asymptomatic volunteers with UTE and ZTE sequences. This raises questions about their nature, i.e., either truly false positives, subclinical lesions, or predictive of future pathologies. The case of the volunteer who developed hindfoot pain one year after the MRI, which showed abnormalities on UTE and ZTE but not on PD-WI, is thought-provoking. It suggests that these sequences might be capable of detecting early changes before the onset of clinical symptoms. The detection of abnormalities in healthy volunteers also highlights the need for caution in interpreting findings on these highly sensitive sequences. Abnormal findings of the Achilles tendon in asymptomatic patients are not rare. Mild increased intra-tendon SI and slightly enlarged tendons can be demonstrated on PD-WI in about two-thirds of asymptomatic patients, peritendonitis and pre-Achilles edema in a third of them, and significant liquid in the retrocalcaneal bursa [21]. Moreover, Achilles spurs have been reported in about 10–18% of patients between 20 and 29 years and in 37–45% of patients older than 80 years in a random population from a trauma clinic [22]. Furthermore, the higher rates of doubtful or pathological findings observed with UTE and ZTE compared to PD-WI, particularly in the SCa and ECa, emphasize the importance of establishing clear criteria for distinguishing between normal variations and truly pathological changes. This would be crucial to avoid overdiagnosis in clinical practice.

It is also important to note that MRI remains a second-line imaging modality, typically performed after CDUS. MRI is reserved for patients with complex symptoms, overlapping mechanical and inflammatory features, or when costly treatments are being considered for suspected rheumatologic disease with inconclusive CDUS findings. Therefore, before revising the roles of CDUS and MRI, further technical advances in MRI are needed—which is the focus of this study. These advances must then be validated in pathological settings and prospectively compared with the current standard (CDUS) in terms of diagnostic accuracy and cost-effectiveness. This will be the objective of future research.

Other future investigations could comprise longitudinal studies following a cohort of healthy volunteers over an extended period and would be invaluable in assessing the predictive value of abnormalities detected on UTE and ZTE. Direct comparisons between symptomatic patients (either with spondylarthropathies or trauma) and healthy volunteers could help establish more precise diagnostic criteria and improve our understanding of the pathophysiology of entheseal disorders.

Our study has limitations. First, as the study involved in vivo imaging in volunteers, no histopathological confirmation of abnormal findings could be performed. Second, as our aim was to depict normal anatomy, we did not include symptomatic patients, but this has been planned in future studies, helped by the knowledge of normal anatomy. Third, our study was focused on a qualitative assessment of the fibrocartilages as it would be achieved in clinical practice and not on a quantitative assessment of the SI in the body of the Achilles tendon, which has already been performed in past studies [15,23,24,25]. For example, prior work has demonstrated the feasibility of T1 and T2 measurements in cadaveric specimens [15], quantified the bound proton fraction in healthy volunteers and in a patient with psoriasis [23], and assessed relative contrast enhancement on CE-UTE images of the Achilles tendon at different echo times, which was found to be higher in patients with rheumatic diseases compared to controls. Due to the very small sizes of the ECa, SCa, and PCa, such measurements would be at risk of bias and partial volume effect. Fourth, the technical issues encountered with the UTE sequence, resulting in non-availability for 24% of patients, highlight the need for further refinement and vigilance during its acquisition in clinical routine.

## 5. Conclusions

Our study demonstrates that UTE and ZTE sequences significantly improve the visualization of the normal anatomy of the Achilles tendon enthesis in healthy adults compared to conventional MRI. Their ability to reveal subtle changes, even in asymptomatic individuals, suggests potential for earlier detection and monitoring of entheseal pathologies. However, this high sensitivity also poses interpretive challenges, underscoring the need for careful clinical correlation. At this stage of development, our key finding is that UTE and ZTE enable MRI to objectively and comprehensively assess each component of the enthesis organ, which conventional PD-WI cannot achieve. Future prospective studies directly comparing these advanced sequences with CDUS in symptomatic patients are essential to define their diagnostic value and clinical role.

## Figures and Tables

**Table 1:** Characteristics of the MR sequences.

Parameters	T1-WI	PD-WI	ZTE	UTE
Acquisition plane	Sagittal	Sagittal and axial	Sagittal and axial	Sagittal and axial
Dimension	2D	2D	3D	3D
Readout type	Cartesian, turbo spin echo	Cartesian, turbo spin echo	Radial	Cones
Field of view (mm)	150 × 150	180 × 180	80 × 80	90 × 90
Frequency	-	-	320	384
In-plane spatial resolution (mm^2^)	0.30 × 0.36	0.47 × 0.51	0.30 × 0.30	0.30 × 0.30
Slice thickness (mm)	3	3	1.3	1.3
Fat suppression	Fat saturation	Fat saturation	Fat saturation	Fat saturation
Flip angle	-	-	4	17
TE	10.1 ms	42 ms	0	32 µs/7.3 ms
TR	598 ms	2760 ms	0	100 ms
NEX	2	2	3.5	1
Bandwidth (kHz)	200	200	31.30	62.5
Acquisition time	2 min 14 s	4 min 49 s	6 min 22 s	5 min 48 s

Note. Abbreviations: NEX: number of excitations; PD-WI: proton density weighted imaging; TE: echo time; TR: repetition time.

**Table 2:** Comparative analysis of the visibility of the normal anatomy on the Achilles enthesis.

Anatomical Location	Visible on PD-WI	Visible on UTE	Visible on ZTE	*p*-Value ^1^	Post-Hoc Adjusted *p*-Values ^2^		
					PD-WI vs. UTE	PD-WI vs. ZTE	UTE vs. ZTE
Entheso cartilage	1/50 (2)	38/38 (100)	48/50 (96)	<0.0001 ***	<0.0001 ***	<0.0001 ***	1
Sesamoid cartilage	0/50 (0)	35/38 (92.1)	36/50 (72)	<0.0001 ***	<0.0001 ***	<0.0001 ***	0.0269 *
Periost cartilage	0/50 (0)	38/38 (100)	48/50 (96)	<0.0001 ***	<0.0001 ***	<0.0001 ***	1
Entire anatomy	0/50 (0)	35/38 (92.1)	36/50 (72)	<0.0001 ***	<0.0001 ***	<0.0001 ***	0.0269 *

Note. Data are numbers of patients with percentages in parentheses. Abbreviations: PD-WI: proton density weighted imaging. * *p* < 0.05, *** *p* < 0.001. ^1^ Chi-square test. ^2^ McNemar test. Adjustments were performed using the Benjamini–Hochberg method.

**Table 3:** Inter- and intra-observer agreements for the assessment of the visibility of the normal anatomy of the Achilles enthesis.

Location	Sequence	Inter-Observer Agreement (%)	Intra-Observer Agreement (%)	Visibility for Reader-1 ^§^	Visibility for Reader-2 (1) ^§^	Visibility for Reader-2 (2) ^§^
Periost cartilage	PD-WI	56 (41.3–69.7)	70 (55.2–81.7)	0/50 (0)	22/50 (44)	11/50 (22)
	UTE	97.4 (84.6–99.9)	94.7 (80.9–99.1)	38/38 (100)	37/38 (97.4)	37/38 (97.4)
	ZTE	96 (85.1–99.3)	100 (91.1–100)	48/50 (96)	50/50 (100)	50/50 (100)
Sesamoid cartilage	PD-WI	92 (79.9–97.4)	88 (75–95)	0/50 (0)	4/50 (8)	4/50 (8)
	UTE	84.2 (68.1–93.4)	92.1 (77.5–97.9)	35/38 (92.1)	35/38 (92.1)	38/38 (100)
	ZTE	68 (53.2–80.1)	94 (82.5–98.4)	36/50 (72)	46/50 (92)	47/50 (94)
Entheso cartilage	PD-WI	94 (82.5–98.4)	90 (77.4–96.3)	1/50 (2)	2/50 (4)	3/50 (6)
	UTE	92.1 (77.5–97.9)	94.7 (80.9–99.1)	38/38 (100)	35/38 (92.1)	37/38 (97.4)
	ZTE	92 (79.9–97.4)	88 (75–95)	48/50 (96)	48/50 (96)	46/50 (92)
Entire anatomy	PD-WI	98 (88–99.9)	96 (85.1–99.3)	0/50 (0)	1/50 (2)	1/50 (2)
	UTE	81.6 (65.1–91.7)	86.8 (71.1–95.1)	35/38 (92.1)	32/38 (84.2)	37/38 (97.4)
	ZTE	66 (51.1–78.4)	88 (75–95)	36/50 (72)	45/50 (90)	45/50 (90)

Note. Agreements are given as percentages with 95% confidence intervals. ^§^: The numbers in parenthese correspond to the first and 2nd reading by the Reader-2.

**Table 4:** Abnormal findings described in the consensus reading according to the MR-sequence and the anatomical location within the Achilles enthesis.

Location	Sequence	True Negative	Abnormal Findings		*p*-Value ^§^
			Doubtful	Pathological	
**Periost Cartilage**	UTE	35/38 (92.1)	1/38 (2.6)	2/38 (5.3)	0.2818
	ZTE	45/50 (90)	2/50 (4)	3/50 (6)	
	PD-WI	50/50 (100)	0/50 (0)	0/50 (0)	
**Sesamoid Cartilage**	UTE	33/38 (86.8)	3/38 (7.9)	2/38 (5.3)	0.0045 **
	ZTE	41/50 (82)	6/50 (12)	3/50 (6)	
	PD-WI	49/50 (98)	0/50 (0)	1/50 (2)	
**Entheso Cartilage**	UTE	29/38 (76.3)	4/38 (10.5)	5/38 (13.2)	0.0746
	ZTE	33/50 (66)	10/50 (20)	7/50 (14)	
	PD-WI	49/50 (98)	1/50 (2)	0/50 (0)	

Note. Abbreviations: PD-WI: proton density weighted imaging. Data are numbers of patients with percentages in parentheses. ^§^ Tests are Chi-square tests. ** *p* < 0.005.

## Data Availability

Raw data and R scripts from the study are available from the corresponding author (Amandine Crombé) upon reasonable request.

## Abbreviations

BMI	Body mass index
CI	Confidence interval
CDUS	Color Doppler ultrasonography
ECa	Entheso cartilage
IQR	Interquartile range
PCa	Periost cartilage
PD	Proton density
RUFIS	Rotating ultra-fast imaging sequence
SCa	Sesamoid cartilage
SD	Standard deviation
SI	Signal intensity
SNR	Signal-to-noise ratio
T2	Transverse relaxation time
UTE	Ultra-short echo time
WI	Weighted imaging
ZTE	Zero echo time

## Appendix A. Summary of Optimizations Included in the ZTE and UTE Sequences Developed by GE HealthCare and Provided to the Study

The ZTE sequence, marketed as SILENZ by GE, is an acquisition technique introduced in 2012. Unlike conventional sequences, it directly captures the free induction decay signal through ultra-rapid gradient switching. This method yields strongly proton density-weighted images (with the option to add T1 weighting via an inversion preparation pulse). ZTE is based on the Rotating Ultra-Fast Imaging Sequence (RUFIS) pulse sequence, where radiofrequency excitation occurs after gradient stabilization, theoretically allowing k0 acquisition at TE = 0 ms [11]. Hardware constraints make capturing the central k-space point challenging, but other samples remain consistent with TE = 0 ms. ZTE employs a 3D center-out radial k-space sampling scheme with constant readout gradients, reducing gradient switching and enabling silent imaging [26].

Furthermore, UTE enables ultra-short echo time imaging through radiofrequency excitation before gradient ramping, capturing signals with rapid gradient switching and simultaneous readout. While inherently prone to acoustic noise and potential artifacts, UTE allows diverse k-space trajectories including radial, spiral, and cone-based acquisitions. The implementation proposed here by GE HealthCare utilized a center-out 3D cone trajectory, dividing k-space into multiple cones with twisted radial lines [27]. This approach provides volumetric morphological imaging with 2.5–5 times acceleration compared to conventional 3D radial techniques, optimizing acquisition efficiency and geometric consistency [28].

**Table A1:** Quality assessment of the ZTE and UTE sequences.

Characteristics	ZTE Sequence	UTE Sequence	*p*-Value
**SNR in Achilles tendon**	40.4 [33.9–47.4] (21.8–62)	29.9 (26.8–32.1) (17.9–44.6)	<0.0001 *** ^1^
**SNR in calcaneus**	21.1 [17.6–23.3] (13.1–33.3)	16.1 [14.9–18.9] (9.3–23.7)	0.0001 *** ^1^
**Quality score**			<0.0001 *** ^2^
Poor	3/38 (7.9)	1/38 (2.6)	
Intermediate	19/38 (50)	2/38 (5.3)	
High	16/38 (42.1)	35/38 (92.1)	

NOTE. Data are numbers of patients with percentages in parentheses for categorical variables, or median, interquartile range, and minimum–maximum range for numeric non-normal variables. Abbreviations: SNR: signal to noise ratio. *** *p* < 0.001. ^1^ Paired Wilcoxon test. ^2^ Chi-square test.
